# Abnormal mRNA Expression Levels of Telomere-Binding Proteins Represent Biomarkers in Myelodysplastic Syndromes: A Case-Control Study

**DOI:** 10.4274/tjh.2016.0364

**Published:** 2017-08-02

**Authors:** Baoshan Liu, Rongdi Yan, Jie Zhang, Bin Wang, Hu Sun, Xing Cui

**Affiliations:** 1 Tianjin Medical University General Hospital, Clinic of Traditional Chinese Medicine, Tianjin, China; 2 The Second Affiliated Hospital of Shandong University of Traditional Chinese Medicine, Department of Respiratory Disease, Jinan, China; 3 Affiliated Hospital of Shandong University of Traditional Chinese Medicine, Department of Hematology, Jinan, China; 4 Affiliated Hospital of Shandong University of Traditional Chinese Medicine, Department of Peripheral Vascular Disease, Jinan, China; 5 Shandong Institute of Traditional Chinese Medicine, Department of Pharmacology, Jinan, China

**Keywords:** Myelodysplastic syndromes, Telomere-binding proteins, Reverse transcription-polymerase chain reaction, International Prognostic Scoring System, World Health Organization Prognostic Scoring System

## Abstract

**Objective:**

As evidence was shown that abnormal shortening of telomeres begins to accumulate in myelodysplastic syndrome (MDS) patients, this study was conducted to determine the relationship between the mRNA expression levels of telomere-binding proteins (TRF1/TRF2/TIN2/TPP1/POT1/RAP1) and the risk level in MDS.

**Materials and Methods:**

There were 40 patients with MDS and 40 normal controls in this study. Methods including telomere content assays and quantitative reverse transcription-polymerase chain reaction were used to examine the mRNA levels of TRF1/TRF2/TIN2/TPP1/POT1/RAP1 in patients with MDS.

**Results:**

Compared to the normal group used as a control, the mRNA expression levels of RAP1/POT1/TPP1 of the patients with MDS were decreased, whereas their levels of TRF1/TRF2 and TIN2 were increased. A positive correlation was found between the TRF1, TRF2, and TIN2 mRNA expression levels and the risk level of the International Prognostic Scoring System (IPSS) and the World Health Organization Prognostic Scoring System (WPSS) criteria; however, a negative correlation was found between RAP1/POT1/TPP1 mRNA expression levels and the risk levels of IPSS and WPSS criteria.

**Conclusion:**

Because the reduction of TRF1/TRF2/TIN2 mRNA expression and the increase of RAP1/POT1/TPP1 mRNA expression are closely related to the risk levels of the IPSS and WPSS criteria in MDS, it is thought that these telomere-binding proteins could lead to abnormal telomere length and function, which cause chromosomal abnormalities in MDS. With this evidence, we suggest that those proteins’ mRNA expressions could be used as biomarkers for the assessment of the risk degree of MDS patients.

## Abstract (tr)

**Amaç:**

Miyelodisplastik sendrom (MDS) hastalarında Telomerlerin anormal olarak kısalması ile ilgili kanıtlar giderek artmaktadır. Bu çalışma telomer bağlayıcı proteinlerin (TRF1/TRF2/TIN2/TPP1/POT1/RAP1) mRNA ekspresyon düzeyleri ile MDS risk skorları arasındaki ilişkinin tespit edilmesi amacıyla yapıldı.

**Gereç ve Yöntemler:**

Bu çalışmaya 40 MDS hastası ve 40 normal kontrol dahil edildi. MDS hastalarında telomer içerik tahlilleri ve kantitatif ters transkripsiyon-polimeraz zincir reaksiyonu TRF1/TRF2/TIN2/TPP1/POT1/RAP1 mRNA düzeylerini incelemek için kullanıldı.

**Bulgular:**

Normal grup kontrol olarak kullanıldı, MDS hastalarında RAP1/POT1/TPP1 mRNA ekspresyon düzeyleri azalmış, ancak sırasıyla TRF1/TRF2 ve TIN2 düzeyleri artmıştı. TRF1, TRF2 ve TIN2 mRNA ekspresyon düzeyleri ile Uluslararası Prognoz Skorlama Sistemi (UPSS) ve Dünya Sağlık Örgütü Prognoz Skorlama Sistemi (DPSS) arasında pozitif korelasyon tespit edildi, RAP1/POT1/TPP1 mRNA ekspresyon düzeyleri ile UPSS ve DPSS kriterleri arasında negatif bir korelasyon bulundu.

**Sonuç:**

MDS’de TRF1/TRF2/TIN2 mRNA ekspresyonunun azalması ve RAP1/POT1/TPP1 mRNA ekspresyonunun artışı ile UPSS ve DPSS kriterleri arasında yakın bir ilişki bulunması, bu telomer-bağlayıcı proteinlerin MDS’de kromozomal anormalliklere sebep olabilecek anormal telomer boy ve fonksiyonları oluşmasına yol açabileceğini düşündürmektedir. Bu nedenle biz bu proteinlerin mRNA ekspresyonlarının MDS hastalarının risk değerlendirmesinde biyobelirteç olarak kullanılabileceğini düşünmekteyiz.

## INTRODUCTION

Chromosome stability is controlled by telomeres, which are regions of repetitive DNA sequences (TTAGGG) at the end of each chromosome regulating end-to-end fusions and recombination. The word “clock” is frequently used to describe telomeres because they can manage the cellular lifespan [1]. Many studies have indicated that telomere length will not change in >80% of tumor cells, which can make themselves immortal by expressing telomerase to sustain the telomere length [2]. Shelterin, which can regulate telomere length and protect telomere function, is an important protein-binding complex composed of telomere repeat factor-1 (TRF1) and -2 (TRF2), protection of telomeres-1 (POT1), TRF2-interacting telomeric protein (RAP1), TRF1-interacting protein 2 (TIN2), and TIN2-organizing protein (TPP1). There are some similarities between TRF1 and TRF2, including their sequence and organization, and they and POT1 can influence the telomeric DNA. The promotion of genetic instability and the increasing of malignancy risk are associated with age-related decline in telomere length [3]. Evidence was shown that abnormal shortening of hematopoietic cells’ telomeres began to accumulate in patients with myelodysplastic syndrome (MDS) and researchers have found that there is a close relationship between abnormal telomere length and poor patient survival [4,5,6,7,8,9,10].

In this study, our goal was to use reverse transcription-polymerase chain reaction (RT-PCR) to detect the mRNA levels of telomere-binding proteins TRF1, TRF2, TIN2, TPP1, POT1, and RAP1 in MDS. We also tried to determine whether there were any relationships between the mRNA levels and the International Prognostic Scoring System (IPSS) and World Health Organization Prognostic Scoring System (WPSS) scores of patients with MDS.

## MATERIALS AND METHODS

### Patients and Controls

In this study, the recorded data of 40 patients (21 men and 19 women; age range: 26-69 years old) and 40 healthy controls (23 men and 17 women; age range: 18-68 years old) from September 2011 to July 2013 were compared (Tables 1 and 2). All of the patients were diagnosed at the same hospital. Patients were divided into cases of refractory anemia (RA), refractory anemia with ringed sideroblasts (RARS), refractory cytopenias with multilineage dysplasia (RCMD), refractory anemia with excess blasts-1 (RAEB-1), and refractory anemia with excess blasts-2 (RAEB-2) based on the 2008 World Health Organization (WHO) diagnosis and classification schemes. None of them had a family history of blood disease and almost all of them presented with different levels of petechiae and fatigue. We obtained bone marrow samples from these patients and from the 40 control subjects. As per the Declaration of Helsinki, informed consent was received from all participants and the study was authorized by the institutional review board of the Affiliated Hospital of Shandong University of Traditional Chinese Medicine.

### RNA Extraction

The TRIzol reagent (Invitrogen, Buenos Aires, Argentina) was used to extract the total RNA of the mononuclear cells obtained from the bone marrow of the patients and the healthy control subjects. RT-PCR was performed with 1X reverse transcription (RT) buffer (Promega, Madison, WI, USA), 200 U/µL Moloney murine leukemia virus RT (Promega), 250 ng/µL random primer (Promega), and 10 mmol/L of each dNTP (Invitrogen). The cDNA synthesis with 1 µg of the total RNA was processed in a total volume of 20 µL for 10 min at 95 °C, 60 min at 37 °C, and 10 min at 95 °C to inactivate the enzyme. The obtained cDNA was stored at -20 °C until use.

### Real-Time qPCR and RT Reaction

**Figure 1:**
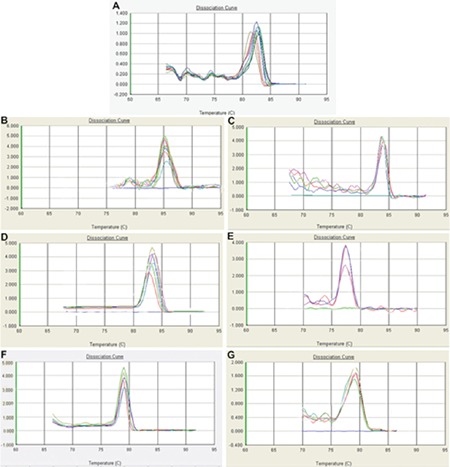
Melting curves of GAPDH (A), TRF1 (B), TRF2 (C), TIN2 (D), RAP1 (E), POT1 (F), and TPP1 (G) polymerase chain reaction products. The straight line indicates a no-template control. GAPDH: Glyceraldehyde 3-phosphate dehydrogenase, TRF1: telomere repeat factor-1, TRF2: telomere repeat factor-2, TIN2: TRF1-interacting protein 2, RAP1: TRF2-interacting telomeric protein, POT1: protecting telomeres-1, TPP1: POT1-and TIN2-organizing protein.

We used the SmartCycler System (Cepheid, Sunnyvale, CA, USA) to test the level of the mRNAs of glyceraldehyde 3-phosphate dehydrogenase (GAPDH), TRF2, TRF1, TPP1, TIN2, and POT1 by using the real-time PCR quantification method. SuperScript III reverse transcriptase (Invitrogen) was used to detect the reverse transcriptions as previously described [11]. Afterwards, the generated cDNA was studied with the Cepheid SmartCycler (software version 2.0c). Based on the manufacturer’s protocol, we designed the PCR primers (Table 3). RT2 Real-Time™ SYBR Green PCR Master Mix (SA BioSciences, Frederick, MD, USA) was used for the proof of gene expression. The 7500 Standard program of the 7500 Fast Real-Time PCR System (Life Technologies, Carlsbad, CA, USA) was used to perform the reactions. Cycling parameters were set as 95 °C for 10 min and then 40 cycles at 95 °C for 15 s, then annealing/extension at 60 °C for 1 min. Based on the PCR cycle number at which the fluorescence emission reached a threshold above the baseline emission, cycle threshold (Ct) values were determined. In the comparative Ct (ΔΔCt) method, we employed GAPDH as an endogenous control to identify the mRNA expression values. The mRNA levels of TRF2, TRF1, TPP1, TIN2, and POT1 were calculated by the following formula: (2 - [ΔCtx - ΔCtr] = 2 - ΔΔCt). The laboratory staff determining the telomere-binding proteins’ mRNA levels were not aware of the patients’ clinical outcomes prior to the statistical analysis. The melting curves of the GAPDH/TRF1/TRF2/TIN2/RAP1/POT1 and TPP1 PCR products are shown in Figure 1.

SPSS 19.0 was used to analyze all of the data. Differences between these data were determined by Student’s t-test with subsequent Bonferroni correction, with p<0.05 considered significant. Data are reported as mean ± standard deviation, and we also used Spearman correlation analysis.

## RESULTS

### Final mRNA Expression Levels in Patients with MDS Compared to Controls

The median age of the normal group and the MDS group was 42.98±13.31 and 44.85±11.46 years, respectively. There was no significant difference between the median ages of these groups. The age distribution of the control group was the same as that of the patient group. The telomere-binding proteins’ mRNA levels were compared between the patients and the healthy controls (Tables 1 and 2). Analyzing these data, significant differences were found between the two groups’ mRNA expression levels of TRF1/TRF2/TIN2/RAP1/POT1 (p<0.01) and TPP1 (p<0.05). There was remarkable variation between the two groups: the mean mRNA expression levels of RAP1/POT1/TPP1 in the patients with MDS were decreased, but the mean mRNA expression levels of TRF1/TRF2/TIN2 were increased.

### Comparison of TRF1/TRF2/TIN2/RAP1/POT1/TPP1 mRNA Expression Levels According to IPSS and WPSS Criteria

**Figure 2:**
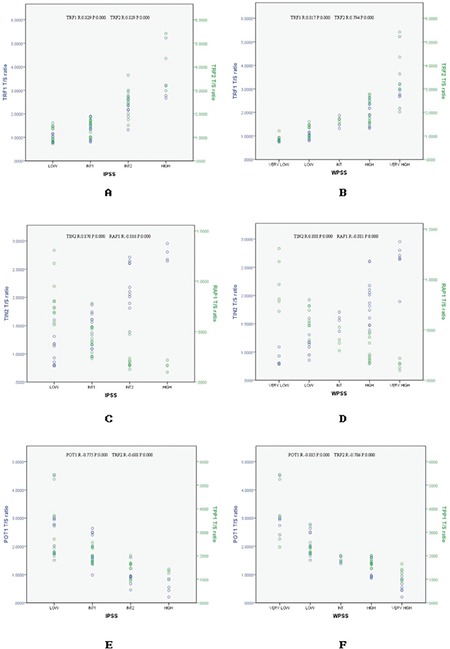
Correlations between the mRNA expression levels of *TRF1/TRF2/TIN2/RAP1/POT1/TPP1* and IPSS/WPSS criteria. TRF1: Telomere repeat factor-1, TRF2: telomere repeat factor-2, TIN2: TRF1-interacting protein 2, RAP1: TRF2-interacting telomeric protein, POT1: protecting telomeres-1, TPP1: POT1: protection of telomeres-1, WPSS: World Health Organization Prognostic Scoring System, IPSS: International Prognostic Scoring System.

The patients in this study had the following 2008 WHO classification subtypes: RA (n=9; 22.5%), RARS (n=8; 20%), RCMD (n=10; 25%), RAEB-1 (n=10; 25%), and RAEB-2 (n=6; 15%). The TRF1/TRF2/TIN2/RAP1/POT1/TPP1 mRNA expression levels were compared with the IPSS and WPSS criteria of the same MDS patient. Combination of the risk scores for IPSS or WPSS criteria stratified the patients into four or five distinctive risk groups, respectively. We found a positive relationship between TRF1/TRF2/TIN2 mRNA expression levels and risk according to IPSS and WPSS criteria. We also found a negative association between RAP1/POT1/TPP1 mRNA expression levels and risk according to the IPSS and WPSS criteria (Figure 2).

## DISCUSSION

Abnormal telomere length regulation exerts a vital influence on hematological malignancies. Genomic instability, as an early event in tumor occurrence, is caused by telomere attrition [12]. Telomere length heterogeneity is seen in all of the MDS subtypes, and decreases of telomere length in MDS are often associated with leukemic transformation and the presence of complex karyotypic abnormalities [4,13].

As a six-polypeptide complex, shelterin is assembled via the binding of the double-stranded TTAGGG repeat binding proteins TRF1 and TRF2, which in turn recruit RAP1, TIN2, TPP1, and POT1 [14,15]. The telomeric DNA is bound by TRF1 and TRF2 in a sequence-specific manner. Shelterin subunits TRF1 and TRF2 are bound in a sequence-specific manner to the double-stranded telomeric DNA, generating a critical platform for recruitment of further shelterin proteins, along with other non-shelterin factors crucial for the maintenance of telomere length and structure. Both TRF1 and TRF2 have the ability to restrict or inhibit telomere elongation by downregulating telomerase [16,17,18]. In diverse eukaryotes, the single-stranded overhang is bound with POT1, and this is fundamental for the protection of chromosome ends and the regulation of telomere length. As another part of the shelterin complex, TPP1 is a binding partner of POT1. Wang and Lei reported the human POT1-TPP1 complex as a telomerase processivity factor [19]. TIN2 is an interesting central shelterin component due to the fact that it connects TPP1/POT1 to the other shelterin components, and furthermore it also stabilizes TRF1 and TRF2 on the duplex telomeric repeat array [20]. TPP1 (previously PTOP/PIP1/TINT1) was identified as a POT1 regulator [21,22,23]. Kibe et al. [21] discovered that telomeres are protected from the repression of the ATR kinases by TPP1-bound POT1a/b. However, some phenomena remain to be understood, such as the interactions between TPP1 and other telomeric proteins, and whether or not it has any functions beyond targeting POT1 and TPP1 that are bound to both POT1 and TIN2 and whether it could tether POT1 to the TRF1 complex. As telomeres can be elongated by the decrease of PIP1 or POT1 levels with short-hairpin RNAs, researchers indicated that telomere length control may be attributed to PIP1 regulation by recruiting POT1 [22]. Studies showed that RAP1 can protect telomere length in yeast [24]. A decrease in RAP1 was found in older cells, especially in an oxidative stress environment. Another interesting study found that if the interaction of RAP1/TRF2 was blocked, the structure of shelterin could be disrupted [25].

As telomere stability and telomere length can be influenced by telomere-binding proteins [26,27,28], a number of experiments were conducted to demonstrate the crucial role of regulating the expression of telomere-binding proteins in cancer and hematological malignancies. POT1-mutated chronic lymphoid leukemia (CLL) cells have numerous telomeric and chromosomal abnormalities that implicate the causative influence of the POT1 mutations for malignant CLL cells [29]. In the early stage of CLL acquisition, telomeric deprotection is the result of both telomere shortening and shelterin (TPP1/TIN2) alteration [30]. This study compared the shelterin complex protein mRNA levels of patients with MDS and healthy volunteers and investigated the relationship between these mRNA expressions and IPSS/WPSS risk in the same patients with MDS.

Two vital conclusions were reached in this study. First, compared with the healthy control group, the mean RAP1/POT1/TPP1 mRNA expression levels of the patients with MDS were significantly decreased, but the mean TRF1/TRF2/TIN2 mRNA expression levels were increased. These data correspond with previously reported telomere lengths in MDS [31,32]. Second, when the TRF1/TRF2/TIN2 mRNA expression levels increased, the risk according to the IPSS or WPSS criteria increased, and when the RAP1/POT1/TPP1 mRNA expression levels increased, the risk according to the IPSS or WPSS criteria decreased. Some significant positive correlations were found between the mRNA expression of RAP1/POT1/TPP1 and that of TRF1/TRF2/TIN2. However, there was a negative relationship between the mRNA expression of RAP1/POT1/TPP1 and that of TRF1/TRF2/TIN2. Upon comparing these results with the findings of other studies, we concluded that the expression of TRF1/TRF2/TIN2 may have a considerable role in the telomere length and dynamics of MDS and the reduction of RAP1/POT1/TPP1 expression may promote the ability of malignant clones of MDS to escape apoptosis and acquire the ability to divide without control.

### Study Limitations

We do not know the mechanism of the reduction of TRF1/TRF2/TIN2 mRNA expression and the upregulation of RAP1/POT1/TPP1 mRNA expression. Furthermore, how to treat these abnormal expressions and regulate the length of telomeres is an interesting question.

## CONCLUSION

After we investigated the relationship of these abnormal expressions of shelterin complex proteins and IPSS and WPSS criteria, respectively, we concluded that those proteins’ mRNA expressions could be used as biomarkers for the assessment of the risk degree of MDS patients.

## Figures and Tables

**Table 1:**
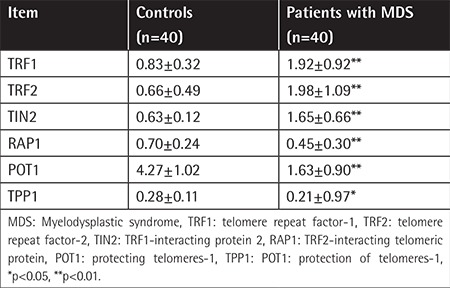
The mRNA expression levels in patients with myelodysplastic syndrome compared to controls.

**Table 2:**
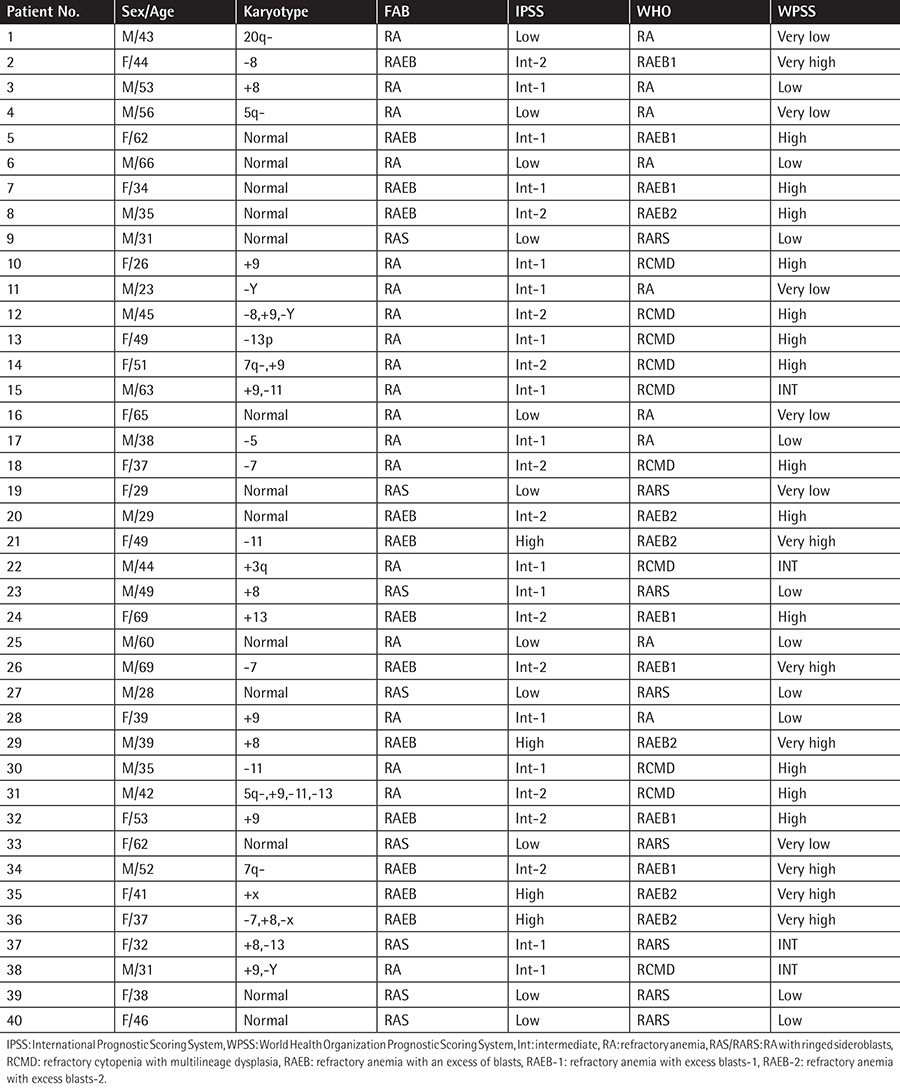
The French-American-British and World Health Organization diagnoses of patients, and the International Prognostic Scoring System and World Health Organization Prognostic Scoring System risk classification.

**Table 3:**
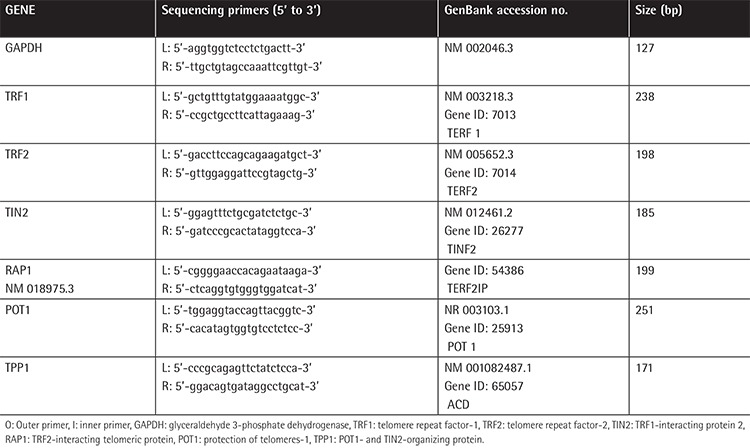
Polymerase chain reaction amplification of mitochondrial DNA genes
